# Frontline work and racial disparities in social and economic pandemic stressors during the first COVID‐19 surge

**DOI:** 10.1111/1475-6773.14136

**Published:** 2023-02-07

**Authors:** Alein Y. Haro‐Ramos, Timothy T. Brown, Julianna Deardorff, Adrian Aguilera, Keshia M. Pollack Porter, Hector P. Rodriguez

**Affiliations:** ^1^ Health Policy and Management University of California Berkeley School of Public Health Berkeley California USA; ^2^ Community Health Sciences University of California Berkeley School of Public Health Berkeley California USA; ^3^ School of Social Welfare Berkeley University of California Berkeley Berkeley California USA; ^4^ Department of Health Policy and Management Johns Hopkins University Bloomberg School of Public Health Baltimore Maryland USA

**Keywords:** determinants of health, health equity, population health, racial/ethnic differences in health and health care, social determinants of health, socioeconomic causes of health

## Abstract

**Objective:**

To assess the magnitude of racial–ethnic disparities in pandemic‐related social stressors and examine frontline work's moderating relationship on these stressors.

**Data Sources:**

Employed Californians' responses to the Institute for Governmental Studies (IGS) poll from April 16–20, 2020, were analyzed. The Pandemic Stressor Scale (PSS) assessed the extent to which respondents experienced or anticipated problems resulting from the inability to pay for basic necessities, job instability, lacking paid sick leave, unavailability of childcare, and reduced wages or work hours due to COVID‐19.

**Study Design:**

Mixed‐effects generalized linear models estimated (1) racial–ethnic disparities in pandemic stressors among workers during the first COVID‐19 surge, adjusting for covariates, and (2) tested the interaction between race–ethnicity and frontline worker status, which includes a subset of essential workers who must perform their job on‐site, to assess differential associations of frontline work by race–ethnicity.

**Data Collection:**

The IGS poll data from employed workers (*n* = 4795) were linked to the 2018 Centers for Disease Control and Prevention Social Vulnerability Index at the zip code level (*N* = 1068).

**Principal Findings:**

The average PSS score was 37.34 (SD = 30.49). Whites had the lowest PSS score (29.88, SD = 26.52), and Latinxs had the highest (50.74, SD = 32.61). In adjusted analyses, Black frontline workers reported more pandemic‐related stressors than White frontline workers (PSS = 47.73 vs. 36.96, *p* < 0.001). Latinxs reported more pandemic stressors irrespective of frontline worker status. However, the 5.09‐point difference between Latinx frontline and non‐frontline workers was not statistically different from the 4.6‐point disparity between White frontline and non‐frontline workers.

**Conclusion:**

Latinx workers and Black frontline workers disproportionately reported pandemic‐related stressors. To reduce stress on frontline workers during crises, worker protections like paid sick leave, universal access to childcare, and improved job security are needed, particularly for those disproportionately affected by structural inequities, such as racially minoritized populations.

**What is known on this topic**

- Pandemic‐related stressors are a key driver of adverse mental health outcomes among the general public during the COVID‐19 pandemic.
- Work is a social‐contextual factor that shapes the degree to which individuals are exposed to pandemic‐related stressors, and frontline work escalates occupational demands and limited workers' agency.
- Because of pre‐existing structural inequities in employment and other determinants of health, Black and Latinx individuals are disproportionately employed as essential frontline workers in precarious jobs.

**What this study adds**

- Black frontline workers reported more pandemic‐related stressors than White frontline workers, resulting from a lack of paid sick leave, unavailability of childcare, job insecurity, reduced wages/work hours, and financial hardships.
- Frontline work did not moderate the degree to which Latinx workers experience pandemic stressors, as Latinxs reported greater pandemic‐related stressors irrespective of having a frontline position.
- Our findings remained even after accounting for individual‐level socioeconomic position and area social vulnerability, underscoring the intersecting roles of racism and precarious work in stressors.

## 1 INTRODUCTION

In 2021, COVID‐19 became the third leading cause of death in the United States (US) and disproportionately reduced life expectancy among Black and Latinx groups,^1^ Along with disproportionate death rates among racially marginalized groups,^2^ the COVID‐19 pandemic has also had deleterious population‐level mental health effects.^3^, ^4^ Recent studies document that pandemic‐related stressors across various life domains are major drivers of adverse mental health outcomes, including depression and anxiety.^5^, ^6^ However, empirical studies assessing pandemic‐related stressors have focused on the general public or people with disabilities and have not examined racial–ethnic differences in stressors. Moreover, pre‐existing economic inequality, occupational characteristics, and other social determinants attributed to racial disparities in COVID‐19 health outcomes may also have led to differential exposure to pandemic‐related stressors.^7^, ^8^ In particular, frontline workers from marginalized racial–ethnic groups may be more vulnerable to experiencing social stressors, which are principal and understudied mechanisms of health disparities.^9^

Because of structural inequities in employment and other determinants of health, certain racial and ethnic groups are disproportionately employed as essential frontline workers in precarious jobs.^10^, ^11^ Sociological studies of the stress process model have established that individuals belonging to structurally disadvantaged groups, including racialized minorities, are more likely to report adverse life events, anticipatory stressors, and chronic strains.^12^, ^13^, ^14^ The social gradient in stress exposure stems from the unequal distribution of opportunities, risks, and resources.^15^ Because racial–ethnic minority groups, like Latinx and Black individuals, have fewer flexible resources (i.e., social connections, knowledge, prestige, and power), they are more vulnerable to a broad range of stressors and are more likely to appraise an event as stressful, even after accounting for socioeconomic position.^13^, ^14^, ^15^, ^16^ Work, especially during the pandemic, is a critical social‐contextual factor that shapes the degree to which individuals are exposed to stressors.^13^ Without explicitly examining work arrangements, the role of work in racial disparities of pandemic‐related stressors may be masked because risks and hazards associated with particular jobs are interrelated with other axes of disadvantage.^17^, ^18^

Job stratification and occupational disadvantage along racial–ethnic lines^19^ shape racially marginalized workers' income, conditions on and off the job, and the ease with which occupations are entered.^20^, ^21^ Black and Latinx workers are disproportionally employed in lower‐status, precarious, and low‐paying jobs, where they are more likely to face employer resistance to implementing safety measures^22^ and exposures to physical hazards and psychosocial risks.^23^ Work experiences during COVID‐19, the types of jobs held (occupational segregation), and the distribution of risks and resources within the workplace (worksite segregation) are also patterned by race and ethnicity.^24^ Given that Black and Latinx workers are substantially more likely than White and Asian workers to be employed in low‐wage jobs characterized by precarious working conditions,^19^ they may disproportionately experience pandemic‐related stressors.

Frontline work during the pandemic escalated occupational demands and limited worker agency over where work could be completed (i.e., home vs. work settings).^25^, ^26^, ^27^ Frontline workers, a subset of essential workers who must leave their homes to perform their job on‐site, have on average lower education levels, have lower wages, and include a larger representation of disadvantaged groups.^28^ The pandemic also highlighted disempowered workers who may not have another option but to work in person due to economic constraints.^18^, ^29^ As such, frontline work may moderate COVID‐19 pandemic‐related stressors and inflict social and economic costs on racial–ethnic minorities due to lower access to psychosocial and economic resources and fewer employer‐based benefits. The lack of work flexibility, limited worker autonomy, and greater demands in jobs where marginalized racial–ethnic groups are overrepresented are potential sources of the diverging experiences for frontline workers of color compared to their White counterparts.^18^

Anticipation of challenges resulting from economic shocks has been previously associated with depressive symptoms^30^; such differential exposure to anticipatory and acute stressors can exacerbate mental health disparities.^15^, ^31^ Social and economic stress can lead to biological (e.g., hypothalamic–pituitary–adrenal axis dysregulation), psychological (e.g., distress), and unhealthy coping (e.g., sleep problems, alcohol use) responses that pose deleterious consequences for physical and mental health.^13^, ^32^, ^33^ In the context of the COVID‐19 pandemic, inequities in risk factors, such as those posed by frontline work, may interact with existing racial inequalities to create diverging experiences that exacerbate stressors for Latinx and Black workers.

To examine whether racial differences in pandemic‐related stressors existed among workers during the first surge of the COVID‐19 pandemic, we analyzed California voter responses to the UC Berkeley Institute for Governmental Studies (IGS) April 2020 poll (*n* = 4795). We hypothesize that Hypothesis 1. Latinx and Black workers will report more pandemic‐related stressors because they have fewer flexible resources than White workers, even after accounting for individual‐level socioeconomic position and area‐level social vulnerability, and Hypothesis 2. Latinx and Black frontline workers will report more pandemic‐related stressors than White frontline workers. Little is known about the distribution of stressors across racial and ethnic groups during the COVID‐19 pandemic. Even before the pandemic, relatively little research explicitly tested whether racial–ethnic differences in experienced and anticipatory stressors exist.^34^ This research is significant because pandemic‐related social stressors could have physical and mental health consequences across the life course and beyond the pandemic.^35^, ^36^, ^37^

## 2 DATA AND METHODS

### 2.1 Data

We analyzed data from the UC Berkeley IGS April 2020 poll of California registered voters. The IGS poll is a recurring web‐based survey of California registered voters to assess public opinion about public policies, economic trends, and social issues. The poll assessed COVID‐19 experiences and was administered in English and Spanish from April 16 to 20, 2020. Email invitations were sent to stratified random samples of California's registered voters and could be completed via cellphone or computer. The overall sample was stratified by age, gender, race–ethnicity, and language to obtain a proper balance of survey respondents across major segments of the state's registered voter population. A total of 150,000 respondents were invited to participate in the survey; 11,502 participated, yielding a 7.7% response rate.^38^ This response rate is consistent with other polls of registered voters.^39^ The completion rate was 76.2%, with 8795 people completing the entire survey. Post‐stratification weights were applied to align the sample of registered voters to the population characteristics of the state's registered voters based on age, race–ethnicity, gender, education, California region of residence, and party affiliation.

Because we were interested in the experiences of employed adults, the analytic sample was restricted to participants who reported being employed at the time of the survey (*n* = 4795), excluding students (*n* = 756), retired individuals (*n* = 2006), and unemployed individuals (*n* = 1228). The final analytic sample includes 4795 employed respondents (2665 non‐frontline workers and 2130 frontline workers) across 1068 zip codes (average 4.5 respondents per zip code, range: 1–29) in California. The Institutional Review Board (2020‐04‐13168) at the University of California, Berkeley, approved the research study.

### 2.2 Measures

#### 2.2.1 Dependent variable

The Pandemic Stressor Scale (PSS), a 5‐item composite that assessed social and economic conditions reported as problematic because of the COVID‐19 pandemic, is the study's dependent variable. Because the first surge of the COVID‐19 pandemic was a societal‐level economic and social shock, it disrupted multiple domains of everyday life beyond health.^40^ The PSS assesses social stressors associated with economic security, family needs, and employment. Respondents were asked, “For each of the following, please indicate the degree to which each is a problem that you expect to face– or are already facing–as a result of COVID‐19”: (1) “Not being able to pay for basic necessities (i.e., food, medication, rent/mortgage),” (2) “Losing my job,” “(3) Lacking paid sick leave,” (4) “Not being able to get childcare,” (5) “Reduced wages or work hours.” Each item is scored using a Likert scale in which 0 corresponds to *no problem at all*, 33.3 to *not much of a problem*, 66.6 to a *serious problem*, and 100 to a *very serious problem*. Item scores were averaged to construct the composite measure (range: 0–100; *α* = 0.84), and higher scores indicate more pandemic‐related social stressors. Exploratory factor analysis of the items yielded an eigenvalue of 2.7 with no other factors with eigenvalues greater than 1.0, and the single factor explained 93.52% of the variation, supporting the use of the composite measure. Factor loadings and Cronbach's alpha and inter‐item correlations for the PSS are presented in Tables A1 and A2, respectively.

#### 2.2.2 Independent variable

Each participant self‐reported their race–ethnicity by answering the following question: “Are you White or Caucasian, Latinx/Hispanic, Black or African‐American, Asian/Pacific Islander, American Indian/Alaskan Native, or a member of another race (i.e., multiracial or other)?” We conceptualize race–ethnicity as social categories that shape the distribution of risks, resources, and opportunities.^41^ It is also important to note that while race and ethnicity are two interrelated yet distinct constructs, for the purposes of this paper, we have combined them into a single construct because we focus on a single racial group (Black people) and a single ethnic group (Latinx people). Respondents identifying as American Indian/Alaskan Native, or multiracial were combined into the “Other Race” category due to the small sample size. Our racial–ethnic categories include Asian/Pacific Islander (PI), Black, Latinx, Other Race, and White. We use “White” as the reference category given this group's larger sample size and historical advantage in social resources and work opportunities relative to racially minoritized groups.

#### 2.2.3 Moderating variable

Frontline work was examined as a moderator of the relationship between race–ethnicity and pandemic‐related stress because of the opportunity for increased worker demand and limited autonomy during the pandemic. We focus on frontline workers rather than essential industries because of the considerable variation among local jurisdictions in the definition of essential industries compared to the relative permanency of frontline positions. Frontline workers are defined as employed individuals who must leave their homes to perform their job on‐site and are in contact with other people regardless of their employment industry. Performing frontline work was assessed using a single item asking employed respondents: “Which best describes your workplace since the California statewide shelter‐in‐place went into effect?” Response options included: (1) “I am able to work from my home,” (2) “I leave home to go to work, but my job involves some contact with other people,” and (3) “I leave home to go to work, and I am in regular contact with other people.” The variable was dichotomized to classify frontline workers as 1 if respondents perform their job at their worksite and have any contact with the public (options 2 or 3) and 0 to indicate individuals who can telework.

#### 2.2.4 Control variables

We controlled for demographic variables including nativity (foreign‐born vs. US‐born), sex (male/female), age (continuous), marital status (single, widowed/separated/divorced, and married/cohabiting), living with a person who is 65 years of age or older (yes or no), and having children in the household (yes or no). Participants' political party affiliations may influence self‐reported pandemic stressors due to the pandemic's politicization^42^; thus, we controlled for political party affiliation (Democrat, Republican, Independent, or Other).

Similar to salient studies,^43^, ^44^ we adjusted for indicators of individual‐level socioeconomic position (SEP) and zip code social vulnerability to determine whether racial disparities in stressors remain. Previous studies indicate that individual‐ and area‐level socioeconomic disadvantage do not fully account for racial differences in an array of health and social outcomes.^43^, ^45^ As a fundamental cause, racism also influences health and social outcomes through personally‐mediated racism and race‐based differences in flexible resources such as prestige, power, and freedom.^46^ For individual‐level SEP, we account for median education level (at least some college vs. bachelor's degree or higher), pre‐pandemic income divided at the approximate median income for a single earner in California (<$59,999 or ≥$60,000), and work industry. We classified work industry categories as (1) health care (all occupations), (2) service‐based, manual, or blue‐collar (hotel and hospitality, retail, agriculture, transportation and utility, construction, manufacturing, delivery services, personal care services, restaurants/bars, janitorial, and landscaping), and (3) professional, white‐collar (professional and business, informational technology, finance and accounting, government services, and education). Frontline workers in the health care industry may have unique experiences from other sectors, so this category was maintained separately. Zip code social vulnerability was assessed using the CDC's Social Vulnerability Index (SVI).^47^ The SVI comprises four indices (i.e., socioeconomic status, household composition and disability, minority status and language, and housing type and transportation) to construct a composite measure of community susceptibility in the occurrence of a societal shock or emergency.^48^

### 2.3 Statistical analysis

Descriptive statistics were conducted to examine racial–ethnic differences in pandemic‐related stressors, measured by the PSS, and each covariate included in our final model. Survey weighted Student's *t*‐tests were used for continuous variables and chi‐square for categorical variables. To test Hypothesis 1, we use a series of two‐level mixed‐effects generalized linear regression models (link: identity, family: Gaussian) to estimate whether Black and Latinx workers report more pandemic‐related stressors, accounting for individual‐level SEP and social vulnerability. To test Hypothesis 2, we include an interaction term that assesses whether frontline work moderates racial disparities in PSS. Multilevel models decompose individual effects from the geographic variation of COVID‐19 impacts, the unequal distribution of risks, and differences in local governmental responses, which may affect the degree to which individuals experience pandemic stressors. Level 2 includes zip codes, and level 1 includes individuals living in each zip code. Further, generalized linear models are particularly useful when the outcome is not normally distributed.^49^, ^50^

We used an incremental, nested approach to estimating regression models. In Model 1, we adjust for key demographics (i.e., sex, age, and nativity) and control variables, including marital status, living with a person over 65 years of age, living with children, and political party affiliation. In Model 2, we add an indicator for performing frontline work, and in Model 3, we adjust for SEP (work industry, income, and education). In Model 4, our main model to test Hypothesis 1, we subsequently adjust for zip code SVI. Model 5, which tests Hypothesis 2, includes an interaction between performing frontline work and race–ethnicity to determine whether performing frontline work has a more pronounced association with pandemic stressors among Black and Latinx essential workers. Model 5 allows us to account for individual‐ and area‐level socioeconomic factors to estimate the remaining role of racism in racial and ethnic inequities in pandemic social stressors among frontline and non‐frontline workers.

To assess the robustness of the interaction and main effect in Model 5 to the exclusion of zip code social vulnerability, we conducted sensitivity analyses in which we excluded the SVI from the model but retained individual‐level SEP. Results of sensitivity analyses are shown in the appendix and briefly summarized at the end of the results section.

We use the margins command to estimate the predicted PSS scores for each racial/ethnic group by frontline worker status based on Model 5. Post‐stratification weights were applied in the descriptive and regression analyses to align the sample to the state's registered voter population. We estimated the variance inflation factor to assess collinearity among covariates in our model and compared each model's AIC to assess model fit. We used 2‐sided statistical tests and considered *p* < 0.05 statistically significant for all analyses. Data were analyzed using Stata 15.0.

## 3 RESULTS

### 3.1 Descriptive analysis

Respondent characteristics, summarized by race–ethnicity, are presented in Table 1. Racial–ethnic groups significantly differed on all study variables except for living with someone over 65. Latinxs comprised a higher share of frontline workers (58.3%) than Whites (38.7%) and other racial–ethnic categories (46.7% of Blacks, 41.4% of Asians, and 49.7% of Other Race). Latinxs were also more likely to work in manual, service, or blue‐collar jobs (51.4%). Black (39.3%) and Latinx (47.1%) respondents were more likely than their White counterparts (20.2%) to report an annual income of less than $60,000. The average PSS score was 37.34 (SD = 30.49; in‐sample range: 0–100); White respondents had the lowest PSS score (29.88, SD = 26.52), and Latinxs had the highest score (50.74, SD = 32.61).

**TABLE 1:** Weighted characteristics of the study sample by race–ethnicity, (*n* = 4795).

Characteristics	White	Asian	Black	Latinx	Other	Total		
	*N* = 2531	*N* = 652	*N* = 300	*N* = 1047	*N* = 265	*N* = 4795		
	%	%	%	%	%	*n*	%	*p*‐value
PSS score, mean (SD)	29.88 (26.5)	43.25 (31.9)	42.87 (32.2)	50.74 (32.6)	34.79 (29.7)	37.34 (30.5)		<0.001
Age, years, mean (SD)	46.45 (14.7)	40.73 (12.4)	42.53 (12.9)	39.70 (13.1)	45.55 (13.7)	44.16 (14.3)		<0.001
Education								
Some college or less	43.3	34.3	48.7	65.9	50.4	2289	47.7	
College degree or higher	56.7	65.7	51.3	34.1	49.6	2506	52.3	<0.001
Frontline worker	38.7	41.1	46.7	58.3	49.7	2130	44.4	<0.001
Living with an adult over 65 years	20.0	24.3	23.7	21.6	20.6	1016	21.2	0.11
Foreign‐born	6.4	52.1	12.6	39.2	23.9	1014	21.1	<0.001
Marital status								
Single, widowed, divorced	32.5	39.8	54.8	39.0	34.5	1747	36.4	<0.001
Married/cohabiting	67.5	60.2	45.2	61.0	65.5	3048	63.6	
Political party affiliation								
Democrat	38.2	44.5	51.7	52.5	26.8	2034	42.4	<0.001
Republican	22.5	18.2	6.6	12.6	22.3	899	18.8	
Independent	30.4	31.1	31.8	25.2	33.5	1419	29.6	
Something else	8.9	6.2	9.8	9.7	17.4	443	9.2	
Female	45.7	47.6	55.5	50.6	49.5	2293	47.8	<0.001
Income								
<$59,999	20.2	24.0	39.3	47.1	32.2	1365	28.5	<0.001
≥$60,000	79.8	76.0	60.7	52.9	67.8	3430	71.5	
Children in household	32.6	39.3	44.5	52.5	33.8	1855	38.7	<0.001
Industry								
Health care	10.8	16.7	17.0	10.8	15.0	588	12.3	<0.001
Professional/White Collar	48.0	46.4	35.9	37.7	37.4	2118	44.2	
Manual/Service/Blue Collar	41.2	36.9	47.1	51.4	47.6	2089	43.6	
Standardized social vulnerability index	−0.22 (0.90)	−0.07 (0.98)	0.39 (0.98)	0.60 (1.03)	−0.04 (0.98)	0.00 (1.00)		<0.001

*Note*: Authors' analysis of the April 2020 Institute for Governmental Studies poll data of California registered voters. Differences were assessed using chi‐square tests for categorical variables and *t*‐tests for continuous variables. The numbers indicate the percentage within the racial–ethnic group unless otherwise noted. Unweighted *N*.

Abbreviation: PSS, Pandemic Stressor Scale.

In Table A3, we show the weighted percentage of frontline and non‐frontline workers reporting each of the five stressors (i.e., job insecurity, loss of wages/hours, lack of paid sick leave, unavailability of childcare, and not being able to pay for basic needs) by race–ethnicity.

### 3.2 Multivariate analysis

Table 2 presents the results of the mixed‐effects generalized linear models. Based on the unadjusted model, about 18.6% of the variation in pandemic‐related social stressors can be explained by zip code‐level factors (likelihood ratio test = 36.4, *p* < 0.001), supporting the use of mixed‐effects models. In Model 1, Latinx (*b* = 12.3, *p* < 0.001), Black (*b* = 5.65, *p* < 0.05), and Asian (*b* = 4.59, *p* < 0.05) workers reported more experienced and anticipatory pandemic‐related stressors than White workers, accounting for demographic variables. In Model 2, when we included an indicator for being an essential frontline worker, the coefficients for Black race and Latinx ethnicity attenuated but remained statistically significant. In this model, frontline work was associated with more experienced and anticipatory pandemic‐related stressors (*b* = 10.0, *p* < 0.001) compared to non‐frontline work. In Model 3, the association of Black race (*b* = 4.92, *p* < 0.05) and Latinx ethnicity (*b* = 8.1, *p* < 0.001) with pandemic‐related stressors remained statistically significant when analyses controlled for individual‐level SEP indicators (i.e., education, income, and industry of employment). In Model 4, which also included zip code SVI, the coefficient for Latinx workers was consistent (*b* = 7.56, *p* < 0.001); however, Black workers did not report more pandemic‐related stressors than White workers once zip code SVI was considered.

**TABLE 2:** Multilevel mixed‐effects generalized linear models of the relationship between race–ethnicity and Pandemic Stressor Scale Scores (*n* = 4795).

	(Model 1)		(Model 2)		(Model 3)		(Model 4)		(Model 5)	
	Coeff.	SE	Coeff.	SE	Coeff.	SE	Coeff.	SE	Coeff.	SE
Race–ethnicity (Ref. White)										
Asian	4.59*	(1.97)	4.87*	(1.92)	5.94**	(1.82)	5.81**	(1.81)	3.49	(2.00)
Black	5.65*	(2.70)	5.31*	(2.59)	4.92*	(2.48)	4.52	(2.48)	−0.25	(2.56)
Latinx	12.3***	(1.52)	11.0***	(1.52)	8.10***	(1.54)	7.56***	(1.57)	7.65***	(1.80)
Other	2.76	(2.17)	1.99	(2.19)	1.02	(2.20)	0.89	(2.20)	2.24	(2.42)
Female (Ref. Male)	−0.20	(1.05)	0.76	(1.04)	0.41	(1.01)	0.35	(1.01)	0.41	(1.01)
Foreign‐born (Ref. US Born)	10.2***	(1.46)	9.48***	(1.43)	9.26***	(1.38)	9.28***	(1.38)	9.22***	(1.37)
Age (years)^a^	−0.14***	(0.04)	−0.13***	(0.04)	−0.067	(0.04)	−0.063	(0.04)	−0.061	(0.04)
Married/cohabiting (Ref. Single)	−3.72**	(1.18)	−3.07**	(1.16)	0.57	(1.14)	0.51	(1.14)	0.47	(1.14)
Children in HH (Ref. No children)	8.53***	(1.19)	8.11***	(1.16)	8.14***	(1.12)	8.12***	(1.11)	8.02***	(1.09)
Living with an adult over 65 years (Ref. no 65+ in HH)	4.84***	(1.32)	4.52***	(1.32)	3.59**	(1.28)	3.50**	(1.28)	3.40**	(1.28)
Political party affiliation (Ref. Democrat)										
Republican	−4.50**	(1.56)	−6.10***	(1.55)	−6.95***	(1.53)	−6.94***	(1.53)	−6.96***	(1.52)
Independent	−2.67*	(1.26)	−3.17*	(1.24)	−3.23**	(1.19)	−3.20**	(1.19)	−3.23**	(1.18)
Something else	1.82	(1.98)	0.80	(1.93)	−0.31	(1.92)	−0.36	(1.93)	−0.41	(1.92)
Frontline worker (Ref. Non‐frontline worker)			10.0***	(1.11)	6.28***	(1.19)	6.08***	(1.19)	4.60**	(1.49)
Industry (Ref. Health Care)										
White Collar					1.50	(1.66)	1.39	(1.66)	1.46	(1.67)
Blue Collar					0.28	(1.64)	0.18	(1.64)	0.13	(1.63)
Bachelor's or higher (Ref. Some College or less)					−7.68***	(1.18)	−7.42***	(1.19)	−7.51***	(1.19)
Income > = $60 K (Ref. <$59,999)					−11.5***	(1.44)	−11.2***	(1.45)	−11.2***	(1.44)
Standardized social vulnerability index							1.21*	(0.54)	1.21*	(0.54)
Race–ethnicity × frontline worker (Ref. White frontline worker)										
Asian # frontline worker									6.25	(3.37)
Black # frontline worker									11.0*	(4.92)
Latinx # frontline worker									0.48	(2.69)
Other # frontline worker									−2.40	(4.38)
Constant	30.7***	(1.27)	26.7***	(1.30)	38.9***	(2.43)	38.8***	(2.42)	39.4***	(2.43)
Level 2 variance	111.9***	(13.53)	103.1***	(12.5)	95.7***	(11.48)	94.3***	(11.38)	91.0***	(11.15)
Level 1 variance	698.2***	(17.44)	681.8***	(17.1)	649.9***	(16.43)	649.9***	(16.42)	649.4***	(16.49)
AIC	42900.3		42773.2		42555.4		42552.2		42544.4	

*Note*: Authors' analysis of the April 2020 Institute for Governmental Studies poll data of California registered voters. Associations were assessed using two‐level mixed‐effects generalized linear regression models (link: identity, family: Gaussian), in which registered voters are level 1, and level 2 are zip code. Model 1 adjusts for key demographic (i.e., sex, age, and nativity) and control variables, including marital status, living with a person over 65 years of age, living with children, and political party affiliation. Model 2 adds an indicator for performing frontline work, and Model 3 adjusts for socioeconomic position (work industry, income, and education). Model 4 subsequently adjusts for zip code Social Vulnerability, and Model 5 subsequently includes an interaction between race–ethnicity and frontline worker status. Standard errors in parentheses.

^a^ Mean‐centered variable.

* *p* < 0.05;

** *p* < 0.01;

*** *p* < 0.001, two‐tailed significance tests.

Control variables were also positively associated with pandemic‐related stressors across the regression models, including nativity (being foreign‐born) and having children in the household. Republican/Independent political party affiliation, a college degree or higher, and a pre‐pandemic income of $60,000 or higher were negatively associated with pandemic‐related stress.

### 3.3 Moderation analysis

**FIGURE 1:**
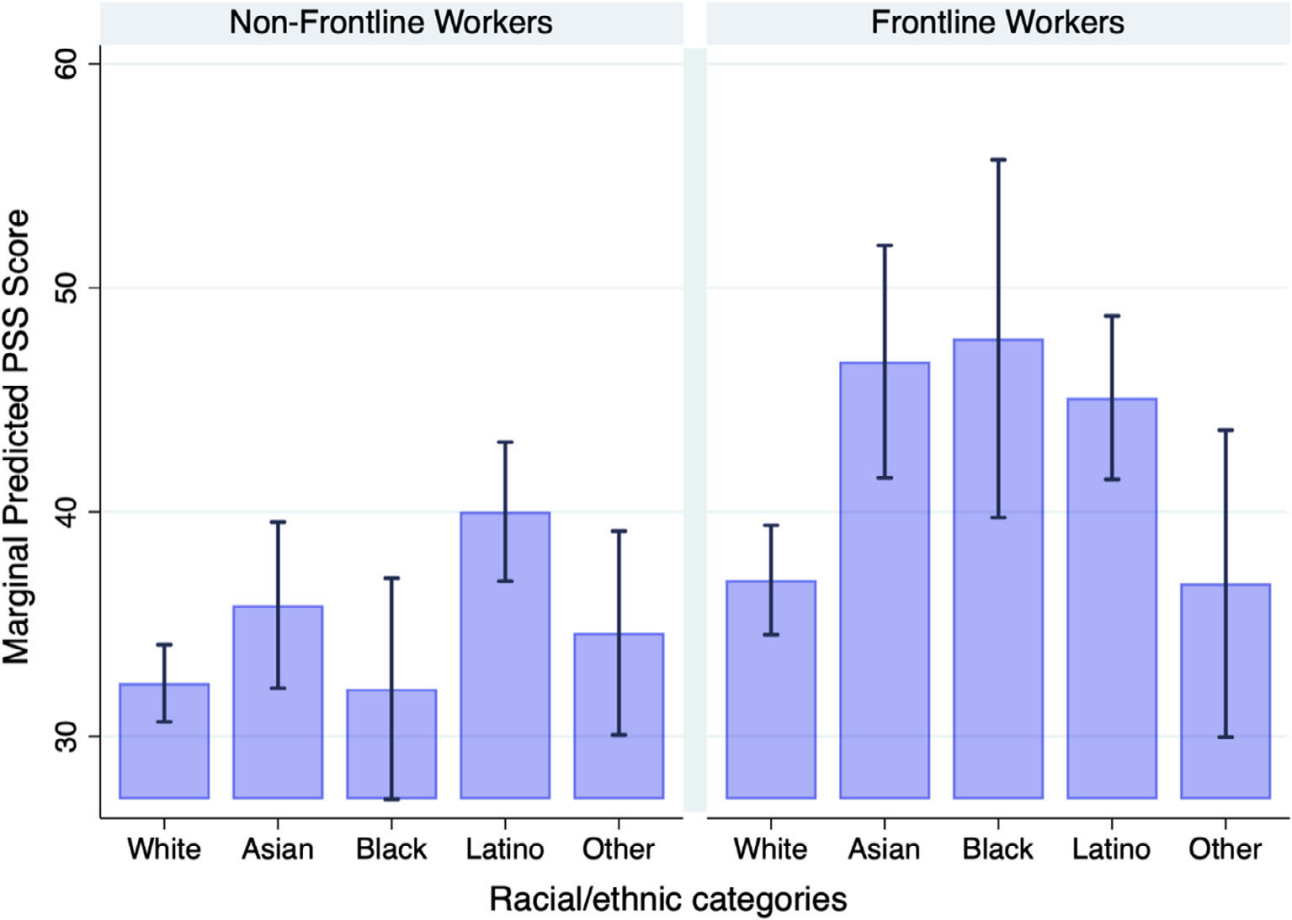
Predicted pandemic stressor scores by race–ethnicity across frontline work based on Model 5. Source: IGS April 2020. The error bars represent 95% confidence intervals around predicted scores. [Color figure can be viewed at wileyonlinelibrary.com]

In Model 5, the interaction between performing frontline work and race–ethnicity, which represents the second partial derivative with respect to both frontline work and race–ethnicity, indicates that frontline work only had a moderating association with pandemic‐related stressors among Black workers. That is, Black frontline workers reported greater pandemic‐related stress (*b* = 11.0, *p* < 0.05) than Black non‐frontline workers, all relative to White frontline and non‐frontline workers. Based on the predicted scores from Model 5 (Figure 1), Black frontline workers (predicted PSS score: 47.73) had an average 10.8‐point higher PSS score than White frontline workers (predicted PSS score: 36.96). While the disparity between Black and White frontline workers appears small in magnitude, if the mean PSS score for Los Angeles County increased by 11 points, it would move the county from the 75th to the 99th percentile in PSS scores among California counties. Table A4 includes all postestimation PSS scores and 95% confidence intervals from Figure 1. We find that the effect of race–ethnicity differs significantly by frontline worker status for both Black (*X* ^2^ (1) = 15.62, *p* < 0.001) and Latinxs (*X* ^2^ (1) = 5.09, *p* = 0.03). In other words, compared to Black and Latinx non‐frontline workers, Black and Latinx frontline workers report an additional 15.62 and 5.09 points on the PSS, respectively.

Latinxs reported more pandemic stressors irrespective of engaging in frontline work (PSS for Latinx frontline workers = 40.01 vs. PSS for Latinx non‐frontline workers = 45.10, *p* < 0.03). The 5.09‐point difference between Latinx frontline and Latinx non‐frontline workers was not significantly higher than the 4.6‐point difference between White frontline workers and White non‐frontline workers.

### 3.4 Sensitivity analyses

In the regression model that includes the interaction between race–ethnicity and frontline work but does not adjust for SVI (shown in Table A5), the direction, magnitude, and significance of the associations were similar to those in the fully adjusted Model 5 reported in Table 2. In this sensitivity analysis, the coefficient of the interaction for Black frontline workers was statistically significant, and the coefficient of the main effect for Black workers remained insignificant.

## 4 DISCUSSION

Differential exposure to stress is a key pathway explaining racial health disparities.^13^, ^32^ However, empirical research about the distribution of social and economic stressors, whether experienced or anticipatory, across racial–ethnic groups remains limited,^14^ especially in the context of the COVID‐19 pandemic. Further, frontline work arrangements during the pandemic became a significant aspect of work that has implications for disparities in experienced and anticipatory stressors among racially minoritized groups compared to more advantaged White workers with greater job flexibility. Our study of California registered voters' experiences of the first COVID‐19 surge provides evidence to fill these gaps. We found that employed Black frontline workers and Latinx workers were more likely to report pandemic‐related stressors during the first COVID‐19 surge than White workers. These relationships are robust to the inclusion of a wide range of individual‐level covariates and zip code social vulnerability. Given the importance of the work environment during the pandemic, we also examined the moderating role of working on the frontline on social stressors. The COVID‐19 pandemic was a societal shock, but pandemic‐related social stressors were experienced and anticipated in non‐random ways that reflect racial stratification in the US.

We found partial support for Hypothesis 1 that Latinx and Black workers would report more pandemic‐related stressors than their White counterparts, even after accounting for individual‐level SEP and social vulnerability. Among Black workers, stressors remained robust to the adjustment of individual‐level SEP but not zip code social vulnerability. When adjusting for SVI, we found that Black workers did not differ in experienced and anticipated stressors from their White counterparts. This finding suggests that area‐level social vulnerability may contribute to Black workers' pandemic‐related stress and highlights the role of local social environments on experiences and anticipation of social and economic stress.

We also found that Latinx workers were more likely to report pandemic stressors even after accounting for individual‐level SEP and zip code‐level social vulnerability. This suggests that other factors, such as precarious work and limited access to flexible resources, such as power, prestige, knowledge, and social connections, may contribute to Latinx workers' disproportionate reporting of pandemic‐related stressors.^46^ Structural racism can govern access to quality jobs and flexible resources that shape social and health outcomes.^46^ Flexible resources during the pandemic include: (1) power (autonomy) and job flexibility to decide whether one wishes to continue working on the frontline without undermining one's financial security, (2) knowing one's occupational rights and having the freedom to execute these without employer retaliation, and (3) possessing social connections that can provide childcare when needed or assistance with economic hardships. Differential access to these flexible resources may be why all Latinx workers and Black frontline workers, compared to White workers, reported more pandemic‐related stressors associated with the first wave of COVID‐19. Future research should focus on modifiable work‐related factors contributing to disproportionate experiences and anticipation of social and economic stress during social crises.

Consistent with Hypothesis 2, we found that Black frontline workers reported more pandemic‐related stressors than White frontline workers. Frontline work did not moderate the degree to which Latinx workers experience pandemic stressors, as Latinxs reported greater pandemic‐related stressors irrespective of having a frontline position. This finding is likely due to the vicarious vulnerability of living in a household dependent on precarious jobs. Latinxs were most likely to report that someone in their household was a frontline worker, which places others in the household at risk of anticipatory pandemic stressors. For example, a national study found that living with at least one worker who cannot work from home is the highest for Latinxs (64.5%) compared to other racial–ethnic groups.^27^

Our results align with recent research on the COVID‐19 pandemic's social impacts on marginalized groups. For example, Perry et al. find that in Indiana, Black individuals, women, those with lower levels of education, and older individuals were more likely to experience pandemic precarity, including food, housing, and financial insecurity.^51^ Our results reinforce the notion that disparities in pandemic precarity reflect pre‐existing structural disadvantages^29^; in this context, racial and ethnic minority groups were disproportionately burdened by pandemic‐related social stressors because of pre‐existing social disadvantages. Consequently, the first COVID‐19 surge in California created additional stressors for already marginalized groups, leaving workers in a perpetual state of susceptibility and unable to respond to future setbacks.

It is important to note that our survey took place a month after the state‐wide California shelter‐in‐place was enacted in April 2020, making our findings unique to a period early in the COVID‐19 pandemic. It is possible that the stressors that frontline workers from racially minoritized groups experienced worsened from when the survey was conducted to early 2021, when COVID‐19 vaccines were not yet available, as workloads increased, and more contagious strains of the virus appeared. With the accumulation of exposure risk, increased workload, and the need to keep working due to economic constraints, it is likely that racial inequities in stress levels may have grown even more pronounced before vaccines became available, particularly for frontline workers employed in low‐wage jobs. Conversely, with the widespread availability of COVID‐19 vaccines in April 2021 and treatment in early 2022 in California, the risk of exposure has been mitigated, and stressors may have subsided for workers with flexible resources to access health care. Nonetheless, racial disparities in COVID‐19 vaccines^52^ and treatment access to Paxlovid^53^ have been documented, and workers from racially minoritized populations may not have adequate access to these health innovations. As such, frontline workers in socially vulnerable communities may continue to work in congregated settings while facing unequal access to vaccines and treatment for COVID‐19. These structural barriers may lead to continued stressors, such as limited access to paid sick leave and economic instability if workers fall sick.

### 4.1 Limitations and future directions

Our findings should be considered in light of some limitations. First, the sampling frame comprises California registered voters, so our results do not generalize to the state's population. Approximately 80% of eligible Californians are registered to vote^54^; the remaining fifth of eligible voters, lawful permanent residents, undocumented immigrants, and individuals convicted of a felony are excluded from our sampling frame. Our findings are likely a conservative measure of racial–ethnic differences in pandemic stressors among workers. Second, our data are cross‐sectional, and we cannot establish temporality or a causal relationship between work and pandemic‐related stressors. Natural experiments of the COVID‐19 pandemic on job‐related stressors may better illuminate the causal effects of racism and frontline work on exposure to stressors. Third, participants self‐reported experienced and anticipatory stressors due to the COVID‐19 pandemic. Future studies integrating biomarkers with survey reports of stressors and mental health may elucidate whether social and economic stressors result in biological wear and tear. Further, while we use post‐stratification weights to align the sample to California's registered voters, we cannot formally compare survey respondents to non‐respondents and cannot rule out non‐response bias. Our findings may also be conservative estimates of racial–ethnic disparities in pandemic‐related stressors, given that the most marginalized participants may not have had the opportunity to respond to this online survey via their cellphone or computer. Finally, we could not examine within‐group heterogeneity to examine the psychosocial burden of the pandemic on immigrant vs. non‐immigrant populations, nor were we able to analyze American Indian/Alaskan Native and multiracial groups as independent categories due to their small sample size. In future research, analyzing within‐group differences by race–ethnicity may help us better understand the antecedents and consequences of pandemic‐related stressors.

## 5 CONCLUSION

Latinx workers and Black frontline workers were more likely to report experienced and anticipatory pandemic‐related stressors during the COVID‐19 first surge. Because the consequences of the pandemic may be long‐lasting, examining the racial differences in social and economic stress exposure emanating from this shock is critical to creating social policies that bolster vulnerable populations against future pandemics and social crises. Given that Black frontline workers and all Latinx workers experienced and anticipated more problems due to a lack of paid sick leave, limited access to childcare, and precarious conditions such as job insecurity, reduced wages or work hours, and financial hardships, public policies should strengthen worker protections that target the unique stressors COVID‐19 has placed on already vulnerable communities. For example, San Francisco enacted the Paid Parental Leave Ordinance (PPLO) in 2017 to fill in gaps in California's Paid Family Leave Act (PFLA). PPLO mandates that employers of firms with more than 20 employees provide supplemental compensation to workers receiving PFLA benefits to obtain 100% of their weekly salary replacement for up to six weeks.^55^ While paid family and sick leave policies have the potential to reduce health disparities by allowing workers to take care of their families without risking their jobs or wages, racial inequities in access to quality, secure employment arrangements systematically limit access to such benefits for racialized minorities employed in the flexible job market or small firms. To achieve health equity, local governments can accelerate these efforts by enacting legislation guaranteeing post‐pandemic relief for poor working families, ample and accessible paid sick leave and childcare, and, most importantly, improved job prospects for racially marginalized workers.

## Supporting information


**Appendix S1.** Supporting information.
