# Supplementary material for: Frontline work and racial disparities in social and economic pandemic stressors during the first COVID‐19 surge
Source: Health Serv Res. 2023 Feb 7;58(Suppl 2):186–97. doi: 10.1111/1475-6773.14136 (PMC10339174; doi:10.1111/1475-6773.14136)
Supplement: Supplementary file 1 — Appendix S1. Supporting information. [file HESR-58-186-s001.docx]

| **Table A1. Factor analysis/correlation of the Pandemic Stressor Scale, (n=4,795)** | | | | |
| --- | --- | --- | --- | --- |
| Method: iterated principal factors | | Retained factors = 3 | | |
| Rotation: (unrotated) | | Number of params = 10 | | |
| **Factor** | **Eigenvalue** | **Difference** | **Proportion** | **Cumulative** |
| Factor 1 | 2.71218 | 2.56843 | 0.9352 | 0.9352 |
| Factor 2 | 0.14375 | 0.09932 | 0.0496 | 0.9847 |
| Factor 3 | 0.04443 | 0.04433 | 0.0153 | 1 |
| Factor 4 | 0.0001 | 0.00029 | 0 | 1.0001 |
| Factor 5 | -0.0002 | . | -0.0001 | 1 |
| LR test: independent vs. saturated: chi2(10) = 9973.75 Prob>chi2 = 0.0000 | | | | |

| **Table A2. Cronbach’s Alpha and Inter-Item Correlations of Pandemic Social Stressors Scale, (n=4,795)** | | | | | | |
| --- | --- | --- | --- | --- | --- | --- |
|  | |  |  |  | Average |  |
|  | |  | Item-test | Item-rest | interitem |  |
| Item | Sign | | correlation | correlation | covariance | alpha |
| *Not being able to pay for necessities (i.e., food, medication, rent/mortgage)* | | + | 0.843 | 0.740 | 655.374 | 0.780 |
| *Losing my job* | | + | 0.830 | 0.714 | 658.251 | 0.786 |
| *Lacking paid sick leave* | | + | 0.801 | 0.666 | 676.211 | 0.800 |
| *Not being able to get childcare* | | + | 0.803 | 0.668 | 676.590 | 0.807 |
| *Reduced wages or work hours* | | + | 0.834 | 0.714 | 642.770 | 0.786 |
| Test scale | |  |  |  | 661.839 | 0.839 |

| **Table A3. Weighted percentage of frontline and non-frontline workers reporting each stressor by race-ethnicity, (n=4,795)** | | | | | | | | | | |
| --- | --- | --- | --- | --- | --- | --- | --- | --- | --- | --- |
|  | **White** | | **Asian** | | **Black** | | **Latinx** | | **Other Race** | |
|  | Non-Frontline | Frontline | Non-Frontline | Frontline | Non-Frontline | Frontline | Non-Frontline | Frontline | Non-Frontline | Frontline |
| ***Not being able to pay for basic necessities (i.e., food, medication, rent/mortgage)*** | | | | | | | | | | |
| No problem at all | 51.24% | 38.26% | 40.11% | 23.75% | 36.47% | 14.15% | 27.13% | 17.96% | 44.92% | 33.78% |
| Not much of a problem | 25.63% | 25.22% | 24.16% | 21.08% | 22.66% | 31.31% | 27.69% | 19.73% | 22.24% | 27.61% |
| Somewhat serious problem | 14.37% | 19.55% | 16.38% | 21.56% | 21.46% | 15.85% | 20.51% | 20.78% | 19.72% | 19.78% |
| Very serious problem | 8.77% | 16.98% | 19.34% | 33.61% | 19.41% | 38.69% | 24.68% | 41.54% | 13.13% | 18.84% |
| ***Losing my job*** | | | | | | | | | | |
| No problem at all | 41.42% | 41.87% | 28.54% | 21.96% | 48.01% | 28.92% | 27.64% | 23.29% | 35.61% | 48.27% |
| Not much of a problem | 28.35% | 26.32% | 29.64% | 22.98% | 12.93% | 15.13% | 23.43% | 18.17% | 28.86% | 15.76% |
| Somewhat serious problem | 18.73% | 15.60% | 19.15% | 12.16% | 23.63% | 18.12% | 18.54% | 15.71% | 17.78% | 9.48% |
| Very serious problem | 11.51% | 16.21% | 22.67% | 42.90% | 15.43% | 37.84% | 30.40% | 42.83% | 17.76% | 26.49% |
| ***Lacking paid sick leave*** | | | | | | | | | | |
| No problem at all | 59.64% | 44.03% | 49.58% | 22.94% | 61.92% | 30.79% | 41.26% | 23.28% | 56.20% | 45.62% |
| Not much of a problem | 19.05% | 20.05% | 22.52% | 20.62% | 12.15% | 13.88% | 17.91% | 18.56% | 17.19% | 15.69% |
| Somewhat serious problem | 10.34% | 15.32% | 13.06% | 21.70% | 7.86% | 9.57% | 15.06% | 14.87% | 11.56% | 14.05% |
| Very serious problem | 10.97% | 20.61% | 14.85% | 34.74% | 18.07% | 45.76% | 25.78% | 43.28% | 15.06% | 24.64% |
| ***Not being able to get childcare*** | | | | | | | | | | |
| No problem at all | 80.71% | 75.79% | 66.39% | 51.15% | 73.69% | 57.08% | 64.86% | 50.77% | 72.88% | 65.53% |
| Not much of a problem | 8.17% | 9.48% | 13.00% | 19.34% | 14.55% | 6.23% | 11.75% | 16.52% | 9.20% | 11.04% |
| Somewhat serious problem | 5.45% | 5.11% | 9.06% | 12.73% | 6.75% | 6.08% | 9.15% | 7.81% | 8.16% | 9.02% |
| Very serious problem | 5.67% | 9.62% | 11.56% | 16.78% | 5.00% | 30.61% | 14.24% | 24.90% | 9.77% | 14.41% |
| ***Reduced wages or work hours*** | | | | | | | | | | |
| No problem at all | 40.78% | 33.37% | 30.47% | 20.88% | 39.03% | 22.61% | 28.81% | 19.38% | 39.05% | 33.49% |
| Not much of a problem | 21.65% | 19.69% | 24.86% | 14.13% | 16.04% | 4.65% | 15.35% | 17.85% | 22.14% | 20.46% |
| Somewhat serious problem | 21.17% | 23.03% | 21.49% | 19.43% | 22.60% | 22.25% | 21.77% | 17.07% | 22.51% | 17.46% |
| Very serious problem | 16.40% | 23.90% | 23.18% | 45.56% | 22.33% | 50.49% | 34.08% | 45.70% | 16.30% | 28.59% |

| **Table A4. Predicted Pandemic Stressor Scale Scores with covariates at the mean by race-ethnicity across frontline work** | | | | | |
| --- | --- | --- | --- | --- | --- |
|  |  | Predicted PSS Score | Std. Error | *p*-score | 95% Conf. Interval |
| Non-Frontline Worker | White | 32.36 | 0.88 | 0.001 | (30.64, 34.08) |
|  | Asian | 35.85 | 1.89 | 0.001 | (32.14, 39.56) |
|  | Black | 32.11 | 2.52 | 0.001 | (27.18, 37.04) |
|  | Latinx | 40.01 | 1.58 | 0.001 | (36.91, 43.11) |
|  | Other | 34.60 | 2.32 | 0.001 | (30.06, 39.15) |
| Frontline Worker | White | 36.96 | 1.25 | 0.001 | (34.52, 39.41) |
|  | Asian | 46.71 | 2.65 | 0.001 | (41.52, 51.89) |
|  | Black | 47.73 | 4.07 | 0.001 | (39.75, 55.71) |
|  | Latinx | 45.10 | 1.86 | 0.001 | (41.45, 48.74) |
|  | Other | 36.81 | 3.49 | 0.001 | (29.96, 43.65) |

Predicted Pandemic Stressor Scale Scores by Frontline Workers vs. Non-Frontline Worker. The results are based on post-estimation results from Table 2, Model 5. Source: IGS April 2020

| **Table A5. Multilevel mixed-effects generalized linear models of the relationship between race-ethnicity and Pandemic Stressor Scale Scores, (n=4,795)** | | |
| --- | --- | --- |
|  | Coeff. | SE |
| **Race-ethnicity** (Ref. White) |  |  |
| Asian | 3.57 | (2.00) |
| Black | 0.24 | (2.56) |
| Latinx | 8.22*** | (1.77) |
| Other | 2.34 | (2.41) |
| **Female** (Ref. Male) | 0.46 | (1.01) |
| **Foreign-Born** (Ref. US Born) | 9.21*** | (1.37) |
| **Age** (years)^a^ | -0.06 | (0.04) |
| **Married/cohabiting** (Ref. Single) | 0.54 | (1.14) |
| **Children in HH** (Ref. No children) | 8.04*** | (1.09) |
| **Living with an adult over 65 years** (Ref. no 65+ in HH) | 3.49** | (1.28) |
| **Political Party Affiliation** (Ref. Democrat) |  |  |
| Republican | -6.97*** | (1.52) |
| Independent | -3.27** | (1.19) |
| Something Else | -0.36 | (1.91) |
| **Frontline Worker** (Ref. Non-frontline worker) | 4.81** | (1.48) |
| **Industry** (Ref. Health Care**)** |  |  |
| White Collar | 1.58 | (1.66) |
| Blue Collar | 0.24 | (1.63) |
| **Bachelor's or Higher** (Ref. Some College or less) | -7.77*** | (1.18) |
| **Income >= $60K** (Ref. <$59,999) | -11.5*** | (1.44) |
| **Race-ethnicity x Frontline Worker** (Ref. White frontline worker) | | |
| Asian # Frontline Worker | 6.39 | (3.38) |
| Black # Frontline Worker | 10.9* | (4.94) |
| Latinx # Frontline Worker | 0.43 | (2.69) |
| Other # Frontline Worker | -2.33 | (4.38) |
| Constant | 39.5*** | (2.44) |
| Level 2 Variance | 92.3*** | (11.25) |
| Level 1 Variance | 649.4*** | (16.50) |
| AIC | 42547.6 |  |
| Note: Standard errors in parentheses; * p<0.05, ** p<0.01, *** p<0.001, two-tailed significance tests. ^a^ Mean centered variable | | |
